# Scleredema-an uncommon cause of swelling in a child-a case report and review of the literature

**DOI:** 10.1186/1756-0500-7-571

**Published:** 2014-08-27

**Authors:** Bikash Shrestha, Arun Kumar Neopane, Rajesh Panth

**Affiliations:** Department of Paediatrics, Nepalese Army Institute of Health Sciences, Shree Birendra Hospital, Swayambhu, Chhauni, Kathmandu, Nepal; Department of Pathology, Nepalese Army Institute of Health Sciences, Shree Birendra Hospital, Swayambhu, Chhauni, Kathmandu, Nepal

**Keywords:** Buschke, Scleroderma, Scleromyxedema, Systemic sclerosis

## Abstract

**Background:**

Body swelling in a child is a common symptom. Apart from systemic causes like renal, hepatic, and cardiac, rarely such a swelling may be caused by dermatologic conditions.

**Case presentation:**

A child presented with swelling of the body which was subsequently diagnosed as scleredema, a rare and benign dermatologic condition. Scleredema can be confused with similar sounding terms like scleroderma and scleromyxedema.

**Conclusions:**

The case is presented to highlight scleredema as a rare cause of body swelling in paediatrics and to differentiate it from similar sounding rare terms like scleroderma and scleromyxedema.

## Background

Swelling of the body is a common problem in paediatric practice. It is generally a manifestation of some underlying systemic disorders which may be renal, hepatic, cardiac, endocrinal, metabolic, anaphylaxis, nutritional and even pulmonary conditions. Rarely, connective tissue disorders and even dermatological causes can lead to body swelling. The investigations for the cause of swelling need to be according to the suspected disorder for prompt diagnosis and successful management. We report a case of body swelling in a child with scleredema. Besides being rare, scleredema can also be confused with diseases having similar terminologies, especially scleroderma and scleromyxedema. This case is reported to familiarize the general paediatrician with the rare entity of scleredema and its differentials, all of which can be among rarer causes of swelling in children.

## Case presentation

A five years old boy, second child of non-consanguineous marriage, born at term with no significant perinatal and past history, presented to our out patient’s department with the complaints of swelling of the whole body. The swelling started insidiously over a course of few days and involved the entire body, including the face, trunk as well as the extremities. There was no associated rash. The child had no preceding symptoms. The swelling was initially progressive but remained static after a few days of onset. The child had no other associated systemic symptoms.

**Figure 1:**
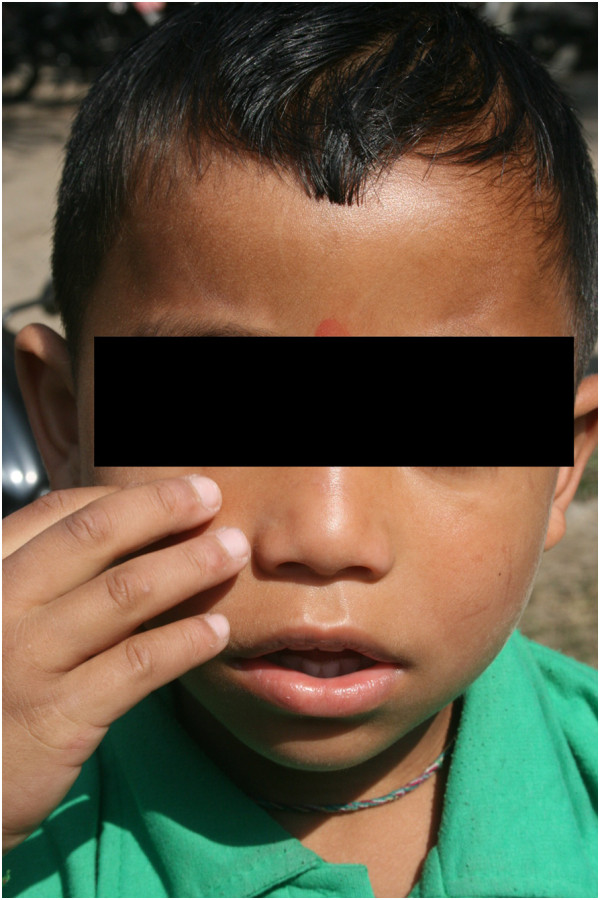
**Picture showing the facial swelling and taut skin.**

**Figure 2:**
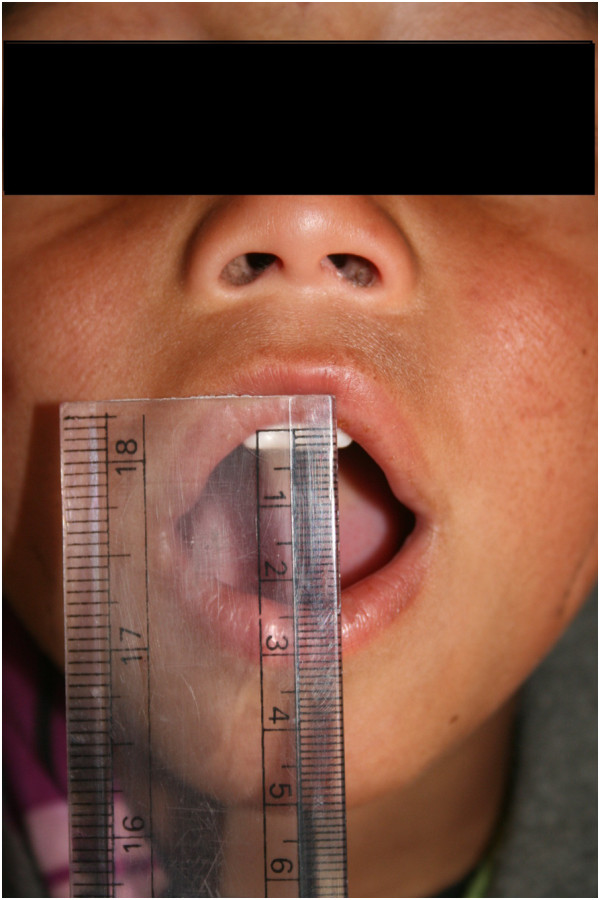
**Picture showing restricted mouth opening.**

On examination, the anthropometry of the child was appropriate for his age. His vital signs, including BP were also within normal limits. Systemic examination was essentially normal. The skin over the face, upper torso, upper and lower limbs excluding the fingers were markedly taut, with non-pitting oedema, woody induration and loss of normal elasticity (Figure 1). There were reduction in mouth opening and the temporomandibular joint mobility (Figure 2). The sensations were intact and there were no other positive findings. Raynaud’s phenomenon was negative. Routine blood investigations, including urinary examination, renal and liver function tests, electrolytes, ASO titers, chest X-ray, ECG, echocardiography and abdominal ultrasonography were normal.

**Figure 3:**
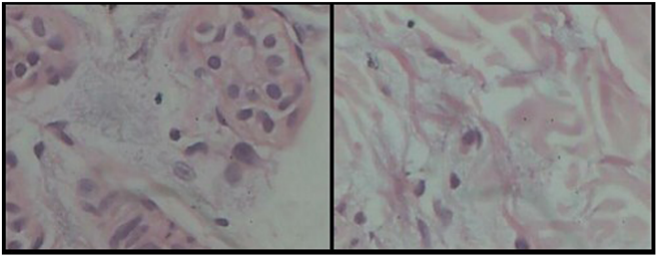
**Biopsy showing smoky bubbly greyish material consistent with mucin deposition between eccrine ducts (left) and collagen bundles (right).** (HE stain, × 400).

The child was admitted for further investigations. Over the days, the swelling remained constant and no other symptoms or signs developed. The common causes including renal, hepatic and cardiac were ruled out. There were no supportive findings to suspect underlying connective tissue disorders. Considering the cutaneous swelling, the possibility of scleredema was kept in mind. In our patient, the fingers were spared and there was no systemic involvement. The findings in our case were suggestive of scleredema. A skin biopsy was undertaken which revealed normal collagen pattern with smoky bubbly greyish materials histologically consistent with mucin deposition between eccrine ducts and collagen bundles (Figure 3). The findings were characteristic of scleredema. Our case was consistent with type I scleredema which was more common in paediatric population. Considering the benign nature of the disease, the parents were counselled about the good prognosis of the disease. The child was discharged after 7 days and has been on regular follow up since then. Over the span of six months, the skin has been gaining its normal elasticity and the child has improved symptomatically.

## Discussion

Scleredema is an uncommon skin disorder which is characterized by non-suppurative, oedematous thickening of the skin, especially the face, neck, shoulders, thorax, proximal extremities but not the fingers [1]. The disease was discovered by Curizo in 1752. Later, Abraham Buschke described the first classical features in an adult patient in 1902 and named it scleredema adultorum to distinguish it from scleredema neonatorum [2]. Subsequently, the disease has been known as scleredema or scleredema of Buschke or scleredema adultorum [3]. The exact incidence, cause and the pathogenesis of the disease are unknown. In 1968, Graff classified the disease into three types-types I, II and III. Type I is the commonest form, and it is especially found in the paediatric population and includes more than 55% of the cases. It typically follows a febrile illness, usually streptococcal throat infection or viral illnesses. This subtype is more common in females and is found in all ages and races. Most cases are below 20 years of age [4, 5]. Type II constitutes around 25% of all cases and is generally progressive and associated with monoclonal gammopathy and multiple myeloma [4]. Type III, also referred to as scleredema diabetocorum or diabetic scleredema, consists around 20% of cases and is a known, rare complication of uncontrolled diabetes mellitus [4, 5]. It is known to occur in 2.5% to 14% of all diabetic cases and found predominantly in men [6]. Scleredema Type I characteristically present with induration of the skin, especially over the face, neck, shoulder girdles and upper extremities but spares the fingers. The mobility of the joints may be reduced. There is absence of Raynaud’s phenomenon which is typically present in scleroderma. In scleredema, there is no organic involvement. The disease is generally self-limiting with active phase being 6–8 weeks and complete resolution within 6 months to 2 years. Occasionally, the disease may be progressive and fatalities have been noted [7, 8]. Rarely, there is involvement of organs like eyes, tongue, oesophagus and the disease may lead to pleural or pericardial or peritoneal effusions [9]. Our patient fitted in type I scleredema, although there was no preceding history of any infection. The treatment modalities for type II and type III is non specific. Various agents that have been tried for the treatment include steroids, methotrexate, cyclosporine, radiotherapy, prostaglandin E1, factor XIII, systemic photochemotherapy with psoralene and ultraviolet A (PUVA), high dose penicillin, d-penicillamine etc [4, 8].

Scleredema is commonly confused with conditions like scleroderma and scleromyxedema. Histologically, scleredema is distinguished by its normal epidermis and thickened dermis, full of swollen collagen fibers and mucin containing clear spaces. Scleroderma is a major cutaneous feature of systemic sclerosis and is typically characterized by pigmented, thickened skin with bilateral, symmetrical involvement and centripetal progression. Histopathologic study shows thin epidermis and thickened dermis with excessive collagen [4, 5, 7, 9]. Scleromyxedema is a papular, waxy, diffuse involvement of the skin of fingers, face, neck and arms. It may be associated with monoclonal gammopathy or multiple myeloma. It is characterized histologically by excessive dermal mucin deposition, fibroblastic proliferation and fibrosis in the absence of thyroid disease [9, 10].

Although rare entities in themselves, miscellaneous skin conditions like scleredema, scleroderma and even scleromyxedema can be the cause of swelling in paediatric population and the primary care paediatrician must be well aware of them so that appropriate investigations can be performed and correct diagnoses made. This is important because in spite of the similarities in terminologies and presentation, the management approach for each of them is quite different.

## Conclusions

The case has been highlighted to familiarize the general paediatric practitioner with an uncommon dermatologic cause of body swelling in children. Despite its rarity and relatively benign nature of scleredema and its diagnostic dilemma with similar conditions like scleroderma and scleromyxedema, with careful history, examination and investigations, the diagnosis can be made and treated accordingly.

### Consent

Written informed consent was taken from the parents for the publication of the case report, along with the accompanying photographs. A copy of the written consent is available for review by the editorial board of the journal.
